# Supplementary material for: Changes in Apolipoprotein A1-Associated Proteomic Composition After Pioglitazone Treatment Versus Weight Loss
Source: Int J Mol Sci. 2025 Nov 3;26(21):10690. doi: 10.3390/ijms262110690 (PMC12608234; doi:10.3390/ijms262110690)
Supplement: Supplementary file 1 [file ijms-26-10690-s001.zip › ijms-3889113-supplementary.pdf]

Supplemental Figures

Supplemental Figure 1. Study Design and Cohort Creation

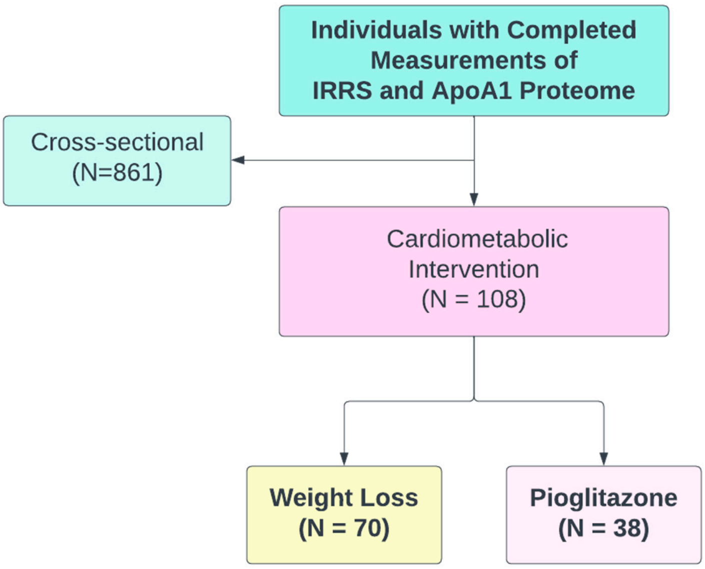

IRRS, insulin resistance risk score.

Supplemental Figure 2. Changes in Weight, SSPG Concentration, and IRRS after Cardiometabolic Interventions

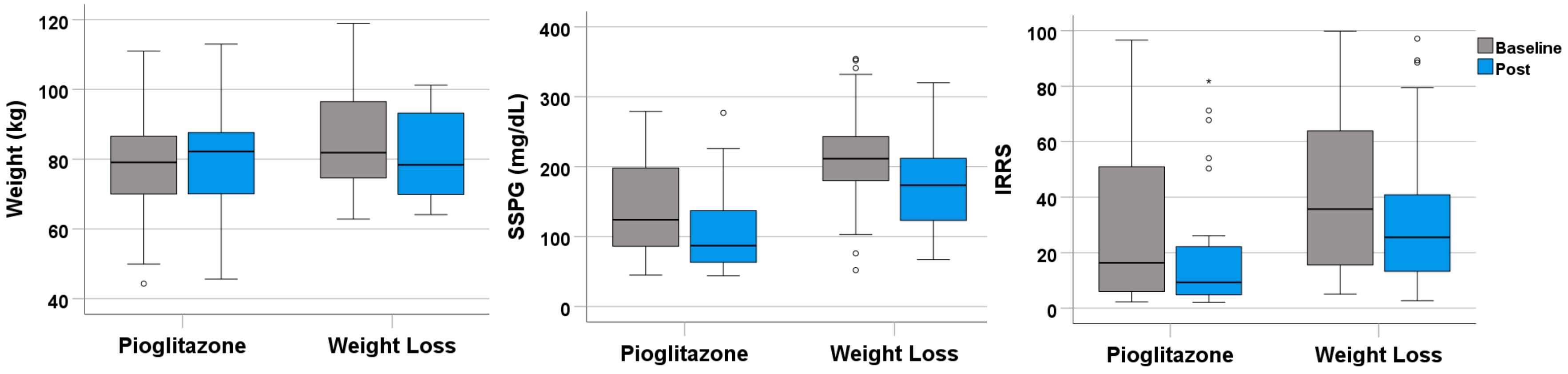

*p<.01 between pre and post intervention within PIO and WL groups using paired t test value for all three outcomes

IRRS, insulin resistance risk score; SSPG, steady-state plasma glucose.

Supplemental Tables

Supplemental Table 1. Changes in Weight, Lipid Levels, and Measures of Insulin Resistance Before and After Cardiometabolic Interventions.

	PIO (n=38)			WL (n=70)			
	**Pre**	**Post**	**p value**	**Pre**	**Post**	**p value**	**p value****
Weight, kg	78.9 (15.3)	79.8 (15.5)*	<0.01	85.6 (14.6)	80.3 (12.1)*	<0.01	<0.001
Total Cholesterol	193 (38)	187 (51)	0.35	193 (35)	175 (39)*	<0.01	0.19
Triglycerides	116 (71)	98 (78)	0.10	153 (91)	108 (38)*	<0.01	0.10
HDL-C	48 (11)	51 (15)	0.20	42 (10)	40 (8)	0.35	0.51
LDL-C	120 (32)	114 (35)	0.25	121 (30)	113 (32)*	<0.01	0.44
Fasting Glucose, mg/dL	95 (11)	92 (10)	0.15	100 (9)	96 (10)*	<0.01	0.43
Fasting Insulin, mU/L	8 (6)	6 (4)*	<0.01	11 (6)	9 (5)*	<0.01	0.94
IRRS, mg/dL	2.8 (1.2)	2.3 (1.0)*	<0.02	3.4 (0.8)	3.1 (0.9)*	<0.01	0.60
SSPG, mg/dL	138 (66)	106 (54)*	<0.01	210 (59)	174 (62)*	<0.01	0.64

Data are mean (SD) unless otherwise noted.
*p<.001 between pre and post intervention within PIO and WL groups using paired t test value
**for change between PIO and WL groups pre and post intervention using unpaired t test
PIO, pioglitazone treatment; WL, weight loss; LDL-C = Low-density Lipoprotein Cholesterol, HDL-C = High-density Lipoprotein; IRRS, insulin resistance risk score; SSPG, steady-state plasma glucose

Supplemental Table 2. Percent Change of ApoA1-Associated Proteins Before and After Cardiometabolic Interventions.

a) PIO

Protein	Percent Change	95% CI
ApoC1	+45.2%	21.5 – 69.1
ApoC2	+41.6%	12.4 – 70.9
ApoC3	+33.1%	1.12 – 65.16
ApoC4	+43.4%	15.3 – 71.6
ApoA2	+36.2%	5.4 – 67.0
ApoA4	+92.9%	55.0 – 130.8
ApoD	+43.4%	13.5 – 73.0
ApoE	+39.2%	4.0 – 74.4
ApoM	+29.1%	7.9 – 50.5
LCAT	+42.1%	13.7 – 70.4
LpPLA2	+79.4%	27.1 – 131.7
PLTP	+49.7%	17.5 – 82.0
PON1	+116.0%	20.9 – 211.0
PON3	+133.3%	34.7 – 191.9

b) WL

Protein	Percent Change	95% CI
ANGT	+54.0%	7.80 – 100.2
ApoA4	+148.0%	48.1 – 248.0
ApoD	+25.2%	6.2 – 44.2
ApoM	+37.6%	6.3 – 68.8
LpPLA2	+75.0%	32.2 – 117.7
PLTP	+48.9%	27.1 – 70.7
PON1	+132.1%	62.5 – 201.7
PON3	+81.6%	33.0 – 130.3

* CI represents the 95% confidence intervals of the percent change. *CI, confidence interval*
